# A Three-Dimensional Evaluation of Skeletal and Dentoalveolar Changes in Growing Class II Patients after Functional Appliance Therapy: A Retrospective Case-Control Study

**DOI:** 10.3390/jcm13051315

**Published:** 2024-02-26

**Authors:** Paolo M. Cattaneo, Annemarie Holm, Augustine K. C. Yung, Stig Isidor, Marie A. Cornelis

**Affiliations:** 1 Melbourne Dental School, Faculty of Medicine, Dentistry and Health Sciences, 720 Swanston Street, Carlton, Melbourne, VIC 3053, Australia; marie.cornelis@unimelb.edu.au; 2 Private Practice, Fisketorvet 4-6, 7.sal, 5000 Odense, Denmark; 3 Private Practice, Hong Kong SAR 999077, China; 4 Private Practice, 8660 Skanderborg, Denmark

**Keywords:** orthodontics, functional orthodontic appliance, cone beam CT, 3D treatment outcome

## Abstract

**Background:** The aim was to assess three-dimensionally mandibular and maxillary changes in growing Class II patients treated with removable functional appliances followed by fixed appliances. **Methods**: Twenty-four Class II patients (age range: 9 to 14, mean: 12.1 ± 1.1 years) treated with removable functional appliances followed by fixed appliances (functional appliance group—FAG) were retrospectively selected and compared to an age-matched control group (CG) treated with fixed appliances only. To be included in the study, pre- and post-treatment CBCT scans had to be available. The CBCTs were used to analyze, in 3D, the changes following treatment and growth. **Results:** Before treatment, overjet (FAG: 9 mm ± 2.8 (mean ± standard deviation); CG: 4 mm ± 1.7), ANB (FAG: 5.7° ± 2.0; CG: 3.2° ± 1.4), and effective mandibular length (FAG: 113.0 mm ± 4.1; CG: 116.6 mm ± 5.9) were statistically significantly different between the two groups. After treatment, overjet (FAG: −6.8 mm ± 2.8; CG: −1.8 mm ± 1.8) and effective mandibular length (FAG: 6.3 mm ± 2.6; CG: 3.9 mm ± 2.6) statistically significantly changed. There was a significant difference in the treatment effect between the FAG and the CG in overjet, ANB, and effective mandibular length. **Conclusions:** The results indicate that functional appliances are effective in correcting Class II malocclusions. The growth modification in the FAG resulted in an increase in mandibular length. Yet, the final length of the mandible in the FAG was smaller when compared to the CG.

## 1. Introduction

Class II malocclusion is characterized by a high prevalence and thus constitutes a significant proportion of patients seeking orthodontic treatment [1,2]. Various factors can contribute to the development of a Class II malocclusion, and their differential diagnosis can help in the selection of the most appropriate treatment approach. Among those factors, mandibular retrognathism shows a prevailing frequency [3,4,5]. In those cases, the use of functional appliances to stimulate mandibular growth has shown to be effective in decreasing overjet and in achieving an Angle Class I canine and molar relationship [6]. Although experiments in animals have demonstrated that skeletal mandibular changes can be produced by positioning the mandible forward [7,8], the effects on humans are more unclear and controversial. While some researchers reported favorable treatment effects on mandibular growth, achieved either as an increased mandibular length [9,10] or as an effective growth of the condyle [11,12], others reported that the effect of functional appliance treatment to the growth of the mandible was not significant [13,14,15,16]. Similar controversies are seen for the maxilla, with some researchers reporting that functional appliances have a restrictive effect on the maxilla [17,18], with others being more skeptical [6,19]. Moreover, the dentoalveolar component changes produced during a successful functional appliance treatment might impact equally or even more than the skeletal effects [20,21].

In the orthodontic literature, the changes following functional appliance therapy have traditionally been assessed by superimpositions of lateral cephalograms. However, this technique has some intrinsic limitations, for example, in assessing growth changes in the transverse plane and with respect to accuracy and reliability [22]. The latter is due to the fact that conventional 2D imaging is subject to magnification, distortion, patient positioning errors and obstruction of critical landmarks by overlapping anatomic structures [23,24]. Additionally, there is inherent examiner bias in the registration process if the examiners are not blinded [25]. Improvements in technology resulted in the development of cone-beam computed tomography (CBCT), which is now readily available since its first introduction in the late 1990s [26,27]. This technique permits accurate 3D images with relatively high resolution and minimal distortion, with visualization of the craniofacial structures in a one-to-one ratio along all the three planes of space [28]. Therefore, it is possible to reliably identify landmarks and to clearly assess, in 3D, the skeletal and dental changes occurring as a result of treatment, without the inherent error of projections typical of lateral cephalograms and the uncertainty in landmarks selection related to the overlapping of bilateral structures [29,30].

Only few studies are available where CBCTs were used to analyze changes in growing Class II malocclusion subjects treated with removable functional appliances [31,32,33].

The aims of the present study were to assess three-dimensionally mandibular and maxillary changes in growing Class II patients treated with removable functional appliances followed by full fixed appliances and to compare them to an age-matched group treated with full fixed appliances only.

The null hypothesis was that there is no additional growth of the mandible or restriction of the maxillary growth following treatment with functional appliances in Class II growing patients, compared to an age-matched control group treated without functional appliances.

## 2. Materials and Methods

The convenience sample used in this retrospective, case-control study was obtained from a list of patients previously treated for Class II malocclusion in the Postgraduate Clinic of the Orthodontic Section at Aarhus University, Denmark, in the period between January 2006 and January 2015.

The inclusion criteria for the functional appliance group (FAG) were as follows:

- Treatment with a removable functional appliance followed by full fixed appliances.
- Overjet of more than 3 mm and a Class II molar relationship (at least half cusp on both sides, or a full Class II molar relationship on one side in case of subdivision).
- Pre- (T0) and post-treatment (T1) CBCT scans and 3D digital models.
- Caucasian origin.

The FAG was compared with an age-matched control group (CG) consisting of skeletal Class I and mild Class II patients treated with fixed appliances selected from the same database of patients during the period between March 2005 and January 2013.

The inclusion criteria for the control group were as follows:

- Class I or mild Class II patients (less than half cusp Class II molar relationship on both sides);
- No use of any kind of functional appliance;
- Limited use of Class II elastics was accepted;
- Pre- (T0) and post-treatment (T1) CBCT scans and 3D digital models.

For both the FAG and CG, the pre- and post-treatment CBCT scans had to have a large field of view (i.e., equivalent to 12″ CBCT scan), so that all the craniofacial structures required for the 3D cephalometric analysis were comprised in the CBCT data-sets. All CBCT scans were taken in supine position and in occlusion (NewTom 3G or 5G, QR s.r.l., Verona, Italy). Scanning parameters: 110 kVp, 5 mA, 0.3 and 0.36 mm isotropic voxel dimension, 18 s of scanning time, 3.6 s of exposure time). All tracings and measurements were performed independently by two operators (A.H. and A.K.C.Y.), who were previously trained and calibrated to identify the relevant 3D landmarks.

The exclusion criteria for both groups were subjects younger than 9 years or older than 14 years.

Written informed consent was obtained from all the patients in agreement with the guidelines of “Sundhedsstyrrelsen” (Danish Health Authority). The data were collected and analyzed with the permission of the Danish Data Protection Agency (#62908).

### 2.1. Image Processing and Analysis

All CBCT scans were reconstructed with an isotropic voxel. Raw data obtained from the CBCT scans were exported in a DICOM format and imported into a specific software (MIMICS 19.0 Materialise, Leuven, Belgium). A new 3D method for cephalometric analysis was developed based on the landmarks and reference lines previously defined by Björk in 2D [34]. Thirty-three conventional landmarks were identified for the cephalometric analysis (Table 1).

**Figure 1:**
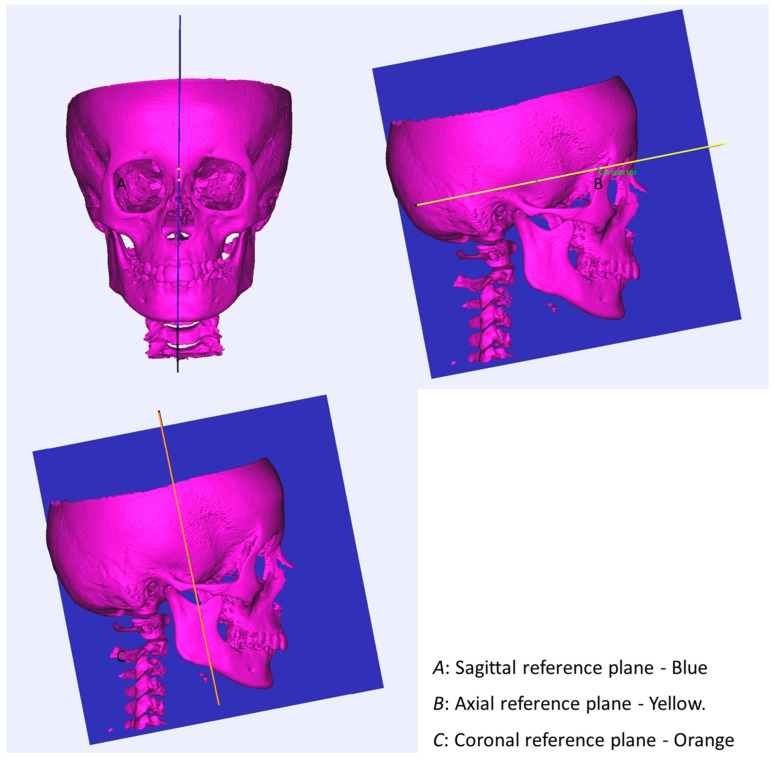
The constructed reference planes are shown.

All mid-sagittal landmarks were identified on the mid-sagittal plane to simulate what is normally performed on lateral cephalograms. The position of each landmark was then checked on the other two orthogonal planes (i.e., the coronal and the axial planes), except for the bilateral points which were identified on the 3D surface and fine-adjusted by checking and relocating them on the coronal, sagittal and axial views. The reference planes were constructed (see definitions and graphical representation in Table 2 and in Figure 1). Specific descriptions of the cephalometric measurements can be found in Table 3. Baseline descriptive data of the two groups were used to display and check the two groups’ characteristics before treatment and to evaluate the skeletal changes.

### 2.2. 3D Digital Model Analysis

The 3D digital models were analyzed in the DDPOrtho software program (Version 1.2_2015; Ortholab, Czestochowa, Poland). Overjet was measured as the distance from the incisal edge of the most proclined upper central incisor to the facial surface of the corresponding central lower incisor.

Overbite was measured as the distance between the incisal edges of the upper and lower central incisors. The molar relationship was assessed according to Angle’s classification.

### 2.3. Statistical Analysis

The statistical analysis was carried out using SPSS (Version 24; SPSS, Chicago, IL, USA). Descriptive measurements including means, standard deviations, and minimum and maximum values of all measured and calculated variables were performed and reported.

Normality was tested with the Shapiro–Wilk test and a Q-Q plot. The data were found to be normally distributed. Therefore, the intra-group differences were assessed using a paired *t*-test, whilst to compare inter-group differences, the independent samples *t*-test was used.

All the FAG and CG subjects were measured independently by two operators (A.H. and A.K.C.Y.), and the technical error of the method in locating the reference points and measuring the variables was assessed. For this purpose, Dahlberg’s formula was used. Bland–Altman plots were also generated and used to assess the mean difference in measurements and to assess the presence of any systematic error between the two operators.

## 3. Results

The technical error of the method revealed that the inter-examiner errors were small for all the measurements (Table 4), and no systematic errors were detected from the Bland–Altman plots.

For the FAG, 24 subjects (14 females, 10 males) with an age ranging from 9 to 14 years (mean age: 12.1 ± 1.1) could be retrieved: 13 out of 24 were skeletal Class II patients and the remaining were skeletal Class I patients. Nine of the patients had a bilateral full Class II molar relationship before treatment. One patient had a bilateral three-quarter cusp Class II molar relationship, and two patients had a half cusp Class II molar relationship. Twelve patients had Class II subdivisions.

For the CG, 18 patients (13 females, 5 males) with an age ranging from 11 to 14 years (mean age: 12.7 ± 0.9) could be retrieved. This group consisted of 16 skeletal Class I patients with different types of malocclusions due to asymmetries in the jaws, ectopic eruption, agenesis, or mild to severe crowding. Two patients presented mild skeletal Class II.

The total mean treatment time was 26.3 ± 6.1 months for the FAG, including phase 1 with removable functional appliance followed by phase 2 with full fixed appliances, while the mean treatment time was 24.2 ± 5.8 months for the CG. This was not statistically different (*p* = 0.74). The phase 1 mean treatment time with removable functional appliances was 10.5 ± 5.4 months.

Treatment modalities in the FAG were distributed as follows: nine Twin Blocks and 15 modified monobloc activators. The baseline data for both groups are reported in Table 5. As expected, the FAG differed significantly from the CG in terms of an increased overjet, enlarged ANB and decreased SNB angles, shorter ramus and effective mandibular lengths, increased Wits appraisal and increased proclination of upper central incisors.

When evaluating the intra-group changes from T0 to T1, a statistically significant difference in all measurements could be seen, except for the SNB angle and the N-S-Ba angle in the FAG. In the CG, significant changes were seen in all measurements except ANB, SNA, SNB and N-S-Ba angles, Wits appraisal and upper right and left central incisor inclinations.

Looking at the inter-group differences, there was a significant difference in the treatment effect between the FAG and the CG in all measurements, except the overbite, SNB and N-S-Ba angle, corpus mandibular length (right), palatal length and lower left central incisor inclination (Table 6).

Concerning the dentoalveolar effects, the FAG had a mean overjet reduction of 6.8 mm ± 2.8 mm which normalized the pretreatment overjet to a mean of 2.1 mm ± 0.5 mm after treatment. Furthermore, an average retroclination of the upper central incisors of 6.6 degrees and an average proclination of the lower central incisors of 8.9 degrees were registered. Looking at the skeletal effects, a positive mean difference of 1.5 mm in ramus length was seen in the FAG compared to the CG; likewise, a 1.3 mm increase in the corpus mandibular length was registered. This resulted in an effective mandibular length increase of 2.4 mm in the FAG when compared to the CG. When looking at the lower anterior facial height, an increase of 1.3 mm was seen in the FAG compared to the CG. The Wits appraisal decreased to 1.6 ± 2.0 mm in the FAG. When comparing the palatal width between the two groups, an increment of 0.6 mm was seen in the FAG compared to the CG.

## 4. Discussion

The material included in this study is quite unique in the sense that, since 2016, CBCT scans are no longer taken for patients where the main problem is an enlarged overjet only, following a revision of the ionizing radiation guidelines for orthodontic diagnosis that became effective at the Department of Dentistry and Oral Health, Aarhus University [35,36].

To the knowledge of the authors, only a few studies [31,32,33] have analyzed 3D skeletal changes in subjects with Class II malocclusion after removable functional appliance therapy. The FAG in the present study consisted of 24 subjects, among which 13 presented with a skeletal Class II. The remaining 11 patients were skeletal Class I patients with an increased overjet and a Class II molar relationship. In an ideal study, the FAG would have consisted of purely skeletal Class II patients, all treated with the same type of removable functional appliance. However, as stated before, this is a retrospective study, with all the related limitations.

The CG consisted of 18 subjects with different types of skeletal pattern: 16 patients were skeletal Class I and 2 were mild skeletal Class II. Fifteen subjects used Class II elastics during their treatment, though for a limited time. Again, ideally, it would have been better if the CG were composed of untreated subjects with a skeletal Class II growth pattern. However, due to ethical reasons, it would be unacceptable to take CBCT scans in untreated Class II subjects at different time points. It is furthermore unacceptable to choose not to treat patients with a Class II malocclusion during their pubertal growth spurt, which is considered the optimal treatment timing [16], and therefore, the CBCT material from the ideal CG will most likely never be available. The fact that many of the CG patients used Class II elastics for a short period of time makes it difficult to completely rule out their influence on growth. Nevertheless, Class II elastics have been shown to act primarily through dentoalveolar modifications with negligible skeletal changes [37,38,39].

In the present study, the treatment time between the two groups only differed by two months on average, with the mean age of the subjects well matched, which allows for the comparison and subtraction of the growth effect. When comparing the baseline characteristics of the two groups, a significant difference in overjet, ANB angle, SNB angle, ramus length, effective mandibular length, Wits appraisal, and upper central incisor inclination was observed. This corresponds very well to what was anticipated. It showed that the FAG generally presented a retrognathic mandible, mainly due to a decreased length of the rami. Additionally, these results illustrated a dentoalveolar difference, where the FAG had dysplastic proclined upper central incisors, which contributed to the increased overjet seen in that group of patients.

When looking at the treatment effect achieved within the FAG, significant changes between T1 and T0 were observed in all measurements, except the SNB angle and the N-S-Ba angle. The fact that a significant treatment effect on the SNB angle was not registered in the FAG group, but that an increase in the effective mandibular length was still observed, differs from earlier studies [31,40,41] where an increase in SNB often is reported after functional appliance therapy. One reason for this could be that the patients experienced a larger degree of posterior rotation of the mandible. This rotation would decrease the SNB angle and increase the lower anterior face height, which was observed in the present study; the increase in total anterior face height and lower anterior face height are common findings after functional appliance therapy, as previously described [42,43]. As presented in the results, in the FAG, a mean overjet reduction of about 7 mm was seen, which corrected the increased overjet and left all subjects with an overjet smaller than 3 mm. A retroclination of the upper central incisors and a proclination of the lower central incisors in the FAG was also observed. Both results are in accordance with earlier studies [43,44].

As mentioned in the introduction, divergent results have been reported, either proving that it is possible to stimulate extra growth of the mandible or disputing it. One explanation for these opposing results could be the differences in treatment timing. Many of the studies where treatment was performed before the growth peak showed small or no skeletal effects, reporting mainly dentoalveolar effects after functional appliance therapy [16,18,45,46,47], whereas studies performed during the subjects’ growth peak showed more skeletal effects, but like in the case of early treatments, they also experienced dentoalveolar effects [18,45]. The importance of treatment timing has been suggested by Baccetti et al. [18] who concluded that growth stimulating treatment with Twin Block showed more skeletal effects when used during the peak of growth. In agreement with Baccetti et al., an increase in growth of the mandible after functional appliance therapy was also observed in the present study. The registered growth corresponded to approximately half of what was lacking from the beginning of treatment. One could argue that the effect of the functional appliance was an acceleration of the growth, which allowed the mandible to catch up on the missing growth. Most of the growth was detected in the rami, which increased its length by 4.5 mm, on average, during the 26.3 months of treatment.

When evaluating the inter-group differences, an increased treatment effect of 1.5 mm ramus length was observed in the FAG compared to the CG. Likewise, a 1.3 mm increase in corpus mandibular length was registered. These two increments led to a mean effective mandibular length increase of 2.4 mm in the FAG compared to the CG. This corresponds to more than 60% increase in growth of the mandible in the FAG compared to the CG. With this growth adaptation, the patients in the FAG nearly caught up with the controls, only lacking less than 2 mm mandibular length. Similar trends were noted by Siara-Olds et al. [43] and Baccetti et al. [18]. These changes indicate that patients treated with removable functional appliances experience more growth of the mandible compared to a treated control group [48]. Furthermore, a reduction in the ANB angle of 1.1 degrees and a reduction in the SNA angle of 0.5 degrees were seen compared to the controls. The decrease in the SNA angle could be due to either a restriction of the maxillary growth or, more likely, to a distal remodeling at the A-point when correcting the initial flaring of the upper incisors. However, the reductions in ANB and SNA angles are minor and can therefore not be seen as clinically relevant. Looking at the transverse relations, a significant increase in palatal width was observed in the FAG compared to the CG. This increased treatment effect of about 0.6 mm could be the result of an active expansion phase during the functional appliance treatment. However, the question of whether this expansion is clinically significant remains open. 

## 5. Conclusions

Treatment with removable functional appliances seems to stimulate growth of the mandible. The additional growth observed in the FAG did surpass the normal growth seen in the treated CG, and this growth stimulation resulted in a nearly normalized length of the mandible in the FAG when compared to the CG. The null hypothesis is therefore assumed to be rejected.

Thus, treatment with removable functional appliances may reduce the severity of the Class II skeletal discrepancy, though a complete correction of the underlying skeletal discrepancy is less likely to be achieved.

Removable functional appliance therapy followed by full fixed appliances is effective in treating dentoalveolar discrepancies in Class II patients and normalizes overjet.

## Figures and Tables

**Table 1:** Hard tissue landmarks used in this study and their definition.

Abbreviation	Landmark	Definition
A-Point	A-Point	Deepest point on the concave outline of the upper labial alveolar process
ANS	Anterior nasal spine	Most anterior midpoint of the anterior nasal spine
Apex L1 L	Apex central lower incisor—left	Apex central lower incisor—left
Apex L1 R	Apex central lower incisor—right	Apex central lower incisor—right
Apex U1 L	Apex central upper incisor—left	Apex central upper incisor—left
Apex U1 R	Apex central upper incisor—right	Apex central upper incisor—right
B-point	B-Point	Deepest point on the concave outline of the lower labial alveolar process
Ba	Basion	The median point of the anterior margin of the foramen magnum
CoL	Condylion—left	Most superior—posterior point of the mandibular condyle—left
CoR	Condylion—right	Most superior—posterior point of the mandibular condyle—right
Crib plate L	Cribriform plate—left	Deepest point of the cribriform plate—left
Crib plate R	Cribriform plate—right	Deepest point of the cribriform plate—right
Crib plate mid	Cribriform plate—mid	Deepest point of the cribriform plate—mid
FML	Foramen mentale—left	Foramen for n. mentalis sin.
FMR	Foramen mentale—right	Foramen for n. mentalis dext.
FPM-L	Foramen palatinum major—left	Foramen for n. palatinus major sin. and a. palatina descendes sin.
FPM-R	Foramen palatinum major—right	Foramen for n. palatinus major dext. and a. palatina descendes dext.
Gn	Gnathion	Most anterior—inferior point on the mandibular symphysis
GoL	Gonion—left	The midpoint at the angle of the mandible—left
GoR	Gonion—right	The midpoint at the angle of the mandible—right
L1L	Incisal edge central lower incisor—left	Incisal edge central lower incisor—left
L1R	Incisal edge central lower incisor—right	Incisal edge central lower incisor—right
L6L	Mesiolingual cusp lower first molar—left	Mesiolingual cusp lower first molar—left
L6R	Mesiolingual cusp lower first molar—right	Mesiolingual cusp lower first molar—right
Me	Menton	The lowest point on the lower border of the mandibular symphysis
N	Nasion	Anterior point of the frontonasal suture
PNS	Posterior nasal spine	Most posterior midpoint of the posterior nasal spine
Pog	Pogonion	Most anterior midpoint of the chin
S	Sella Turcica	Center of sella turcica
U1L	Incisal edge central upper incisor—left	Incisal edge central upper incisor—left
U1R	Incisal edge central upper incisor—right	Incisal edge central upper incisor—right
U6L	Mesiopalatal cusp upper first molar—left	Mesiopalatal cusp upper first molar—left
U6R	Mesiopalatal cusp upper first molar—right	Mesiopalatal cusp upper first molar—right

**Table 2:** Constructed reference planes.

Abbreviation	Plane	Definition
N-S-Ba	Sagittal plane	The longitudinal plane that divides the head into right and left sections
S-crib plate mid-perp to sagittal plane	Axial plane	Plane perpendicular to the sagittal plane going through the stable structures of the cranial base
S-perp to axial plane and sagittal plane	Coronal plane	Plane perpendicular to the sagittal plane and axial plane passing through sella
GoL-Me-GoR	Mandibular plane	Tangent to the lower border of the body of the mandible
U1R-U6R-U6L	Occlusal plane	A plane passing through the occlusal surfaces of the teeth in maximum interdigitation
ANS-PNS and perp to sagittal plane	Palatal plane	Plane perpendicular to the sagittal plane going through the most anterior and posterior point of the hard palate
U6R—perp to sagittal plane and occlusal plane	Wits plane	A vertical plane going through the first upper molar enabling the measurements for the Wits appraisal
L1L-Apex L1L	Mand. incisor inclination L	Plane going through the incisal edge and apex of the left central lower incisor perpendicular to the sagittal plane
L1R-Apex L1R	Mand. incisor inclination R	Plane going through the incisal edge and apex of the right central lower incisor perpendicular to the sagittal plane
U1L-Apex U1L	Max. incisor inclination L	Plane going through the incisal edge and apex of the left upper central incisor perpendicular to the sagittal plane
U1R-Apex U1R	Max. incisor inclination R	Plane going through the incisal edge and apex of the right upper central incisor perpendicular to the sagittal plane

**Table 3:** Cephalometric descriptive measurements.

Measurement	Definition
Corpus mand. length Left	Linear measurement between GoL and Gn
Corpus mand. length Right	Linear measurement between GoR and Gn
Effective mand. length Left	Linear measurement between CoL and Gn
Effective mand. length Right	Linear measurement between CoR and Gn
LAFH	Linear measurement between ANS and Me describing the lower anterior face height
UAFH	Linear measurement between N and ANS describing the upper anterior face height
AFH	Linear measurement between N and Me describing the total anterior face height
Palatal length	Linear measurement between PNS and ANS
Palatal width	Linear measurement between foramen palatinum major left and right
Ramus length (left)	Linear measurement between CoL and GoL
Ramus length (right)	Linear measurement between CoR and GoR
N-S-Ba angle	Angle formed by the intersection of lines SN and SBa, which measures the cranial base angle
ANB angle	Angle formed by the intersection of lines NA and NB, which measures the anterior– posterior relation of the maxilla and the mandible
SNA angle	Angle formed by the intersection of lines SN and NA, which measures the horizontal position of the maxilla in relation to the cranial base
SNB angle	Angle formed by the intersection of lines SN and NB, which measures the horizontal position of the mandible in relation to the cranial base
Wits appraisal	Distance from Wits plane to A-point subtracted the distance from wits plane to B-point
Upper right central incisor inclination	Angle between the palatal plane and the plane for max. incisor inclination—right
Upper left central incisor inclination	Angle between the palatal plane and the plane for max. incisor inclination—left
Lower right central incisor inclination	Angle between the mandibular plane and the plane for mand. incisor inclination—right
Lower left central incisor inclination	Angle between the mandibular plane and the plane for mand. incisor inclination—left

**Table 4:** Error of the method: Dahlberg’s formula.

Measurement	FAG	CG
ANB (°)	0.37	0.28
SNA (°)	0.47	0.57
SNB (°)	0.37	0.46
N-S-Ba (°)	0.62	0.74
Corpus mandibular length left (mm)	0.80	1.08
Corpus mandibular length right (mm)	0.87	1.04
Ramus length left (mm)	1.02	1.15
Ramus length right (mm)	1.09	1.07
Effective mandibular length left (mm)	0.55	0.57
Effective mandibular length right (mm)	0.47	0.58
Palatal length (mm)	1.05	1.40
Palatal width (mm)	0.61	0.52
Total anterior face height (mm)	0.44	0.61
Upper anterior face height (mm)	0.46	0.59
Lower anterior face height (mm)	0.48	0.45
Wits appraisal (mm)	0.46	0.35
Upper right central incisor inclination (°)	1.22	1.29
Upper left central incisor inclination (°)	1.02	1.15
Lower right central incisor inclination (°)	1.27	1.22
Lower left central incisor inclination (°)	0.92	1.09

**Table 5:** Comparison of baseline measurements of functional appliance group and control group.

Measurement	FAG		CG		Comparison	
	Mean	SD	Mean	SD	*p*-Value	Significance
Overjet (mm)	8.9	2.8	4.0	1.7	<0.01	*
Overbite (mm)	4.0	1.7	3.8	1.5	0.45	NS
ANB (°)	5.7	2.0	3.2	1.4	<0.01	*
SNA (°)	80.1	3.7	79.6	4.8	0.95	NS
SNB (°)	74.4	3.4	76.6	4.7	0.04	*
S-N-Ba (°)	132.1	4.4	132	7.0	0.97	NS
Corpus mandibular length (left) (mm)	80.4	4.0	82.7	5.1	0.13	NS
Corpus mandibular length (right) (mm)	80.1	3.4	81.4	4.9	0.31	NS
Ramus length (left) (mm)	49.9	3.0	53.7	4.5	<0.01	*
Ramus length (right) (mm)	50.6	3.2	54.0	4.1	<0.01	*
Effective mandibular length (left) (mm)	112.5	4.0	116.9	5.9	0.01	*
Effective mandibular length (right) (mm)	113.0	4.1	116.6	5.9	0.02	*
Palatal length (mm)	53.2	2.5	51.9	3.5	0.17	NS
Palatal width (mm)	27.5	1.5	27.6	2.3	0.89	NS
Total anterior face height (mm)	107.8	6.6	109.6	6.2	0.36	NS
Upper anterior face height (mm)	49.1	2.6	49.6	2.8	0.52	NS
Lower anterior face height (mm)	61.0	5.7	61.5	4.7	0.77	NS
Wits appraisal (mm)	5.6	2.7	0.5	1.5	<0.01	*
Upper right central incisor inclination (°)	67.3	6.5	72.5	6.6	0.02	*
Upper left central incisor inclination (°)	66.7	5.9	72.0	7.0	0.01	*
Lower right central incisor inclination (°)	85.2	3.4	83.5	6.5	0.27	NS
Lower left central incisor inclination (°)	84.5	3.9	82.3	6.6	0.29	NS

NS. Not significant. * Significant at *p* ≤ 0.05.

**Table 6:** Changes between pre- and post-treatment, and comparison of the treatment effect between the two groups.

Measurement	FAG (T1-T0)				CG (T1-T0)				Comparison of Treatment Effect	
	Mean	SD	*p*-Value	Significance	Mean	SD	*p*-Value	Significance	*p*-Value	Significance
Overjet (mm)	−6.8	2.8	<0.01	*	−1.8	1.8	<0.01	*	<0.01	*
Overbite (mm)	−2.2	1.8	<0.01	*	−2.2	1.6	<0.01	*	0.97	NS
ANB (°)	−1.2	1.5	<0.01	*	−0.1	0.6	0.37	NS	0.01	*
SNA (°)	−0.8	0.8	<0.01	*	−0.3	0.7	0.06	NS	0.06	*
SNB (°)	0.5	1.9	0.18	NS	−0.3	0.9	0.19	NS	0.10	NS
N-S-Ba (°)	−0.1	1	0.52	NS	0.2	1.1	0.38	NS	0.27	NS
Corpus mandibular length (left) (mm)	3.6	1.8	<0.01	*	2.3	1.8	<0.01	*	0.02	*
Corpus mandibular length (right) (mm)	3.8	1.8	<0.01	*	2.8	1.9	<0.01	*	0.09	NS
Ramus length (left) (mm)	4.5	2.2	<0.01	*	3	1.9	<0.01	*	0.04	*
Ramus length (right) (mm)	4	1.9	<0.01	*	2.5	1.8	<0.01	*	0.02	*
Effective mandibular length (left) (mm)	6.3	2.6	<0.01	*	3.9	2.6	<0.01	*	<0.01	*
Effective mandibular length (right) (mm)	6.1	2.6	<0.01	*	3.8	2.2	<0.01	*	0.01	*
Palatal length (mm)	1.8	1.4	<0.01	*	1.3	1.1	<0.01	*	0.24	NS
Palatal width (mm)	1.4	0.7	<0.01	*	0.8	0.5	<0.01	*	<0.01	*
Total anterior face height (mm)	7.1	2.6	<0.01	*	4.6	2.3	<0.01	*	<0.01	*
Upper anterior face height (mm)	2.2	1.3	<0.01	*	1.2	1.1	<0.01	*	0.01	*
Lower anterior face height (mm)	4.8	2.1	<0.01	*	3.5	2	<0.01	*	0.01	*
Wits appraisal	4	2.3	<0.01	*	−0.4	0.9	0.06	NS	<0.01	*
Upper right central incisor inclination (°)	6.4	7.5	<0.01	*	−2	4.9	0.1	NS	<0.01	*
Upper left central incisor inclination (°)	6.9	6.8	<0.01	*	−1.8	6.9	0.3	NS	<0.01	*
Lower right central incisor inclination (°)	−8.9	4.9	<0.01	*	−4.5	6.0	0.01	*	0.01	*
Lower left central incisor inclination (°)	−8.8	5.8	<0.01	*	−5.5	8.0	0.01	*	0.13	NS

FAG. In terms of sagittal jaw relationship, a significant reduction in the ANB angle by −1.1 degrees and of the SNA angle by −0.5 degrees was seen compared to the CG. NS. Not significant. * Significant at *p* ≤ 0.05.

## Data Availability

The data presented in this study are available on request from the corresponding author. The data are not publicly available due to privacy restrictions.

## References

[1] McNamara J.A. (1981). Components of class II malocclusion in children 8–10 years of age. Angle Orthod..

[2] McLain J.B., Proffitt W.R. (1985). Oral health status in the United States: Prevalence of malocclusion. J. Dent. Educ..

[3] Pancherz H., Zieber K., Hoyer B. (1997). Cephalometric characteristics of Class II division 1 and Class II division 2 malocclusions: A comparative study in children. Angle Orthod..

[4] Jacob H.B., Buschang P.H. (2014). Mandibular growth comparisons of Class I and Class II division 1 skeletofacial patterns. Angle Orthod..

[5] Ellis E., McNamara J.A., Lawrence T.M. (1985). Components of adult Class II open-bite malocclusion. J. Oral Maxillofac. Surg..

[6] McNamara J.A., Bookstein F.L., Shaughnessy T.G. (1985). Skeletal and dental changes following functional regulator therapy on class II patients. Am. J. Orthod..

[7] McNamara J.A., Bryan F.A. (1987). Long-term mandibular adaptations to protrusive function: An experimental study in Macaca mulatta. Am. J. Orthod. Dentofac. Orthop..

[8] Woodside D.G., Metaxas A., Altuna G. (1987). The influence of functional appliance therapy on glenoid fossa remodeling. Am. J. Orthod. Dentofac. Orthop..

[9] Marsico E., Gatto E., Burrascano M., Matarese G., Cordasco G. (2011). Effectiveness of orthodontic treatment with functional appliances on mandibular growth in the short term. Am. J. Orthod. Dentofac. Orthop..

[10] de Almeida M.R., Flores-Mir C., Brandao A.G., de Almeida R.R., de Almeida-Pedrin R.R. (2008). Soft tissue changes produced by a banded-type Herbst appliance in late mixed dentition patients. World J. Orthod..

[11] Pancherz H., Ruf S., Kohlhas P. (1998). “Effective condylar growth” and chin position changes in Herbst treatment: A cephalometric roentgenographic long-term study. Am. J. Orthod. Dentofac. Orthop..

[12] Serbesis-Tsarudis C., Pancherz H. (2008). “Effective” TMJ and chin position changes in Class II treatment. Angle Orthod..

[13] Creekmore T.D., Radney L.J. (1983). Frankel appliance therapy: Orthopedic or orthodontic?. Am. J. Orthod..

[14] Gianelly A.A., Brosnan P., Martignoni M., Bernstein L. (1983). Mandibular growth, condyle position and Frankel appliance therapy. Angle Orthod..

[15] Cozza P., Baccetti T., Franchi L., De Toffol L., McNamara J.A. (2006). Mandibular changes produced by functional appliances in Class II malocclusion: A systematic review. Am. J. Orthod. Dentofac. Orthop..

[16] Perinetti G., Primozic J., Franchi L., Contardo L. (2015). Treatment Effects of Removable Functional Appliances in Pre-Pubertal and Pubertal Class II Patients: A Systematic Review and Meta-Analysis of Controlled Studies. PLoS ONE.

[17] Vargervik K., Harvold E.P. (1985). Response to activator treatment in Class II malocclusions. Am. J. Orthod..

[18] Baccetti T., Franchi L., Toth L.R., McNamara J.A. (2000). Treatment timing for Twin-block therapy. Am. J. Orthod. Dentofac. Orthop..

[19] Sidhu M.S., Kharbanda O.P., Sidhu S.S. (1995). Cephalometric analysis of changes produced by a modified Herbst appliance in the treatment of Class II division 1 malocclusion. Br. J. Orthod..

[20] Wieslander L., Lagerstrom L. (1979). The effect of activator treatment on class II malocclusions. Am. J. Orthod..

[21] Robertson N.R. (1983). An examination of treatment changes in children treated with the function regulator of Frankel. Am. J. Orthod..

[22] Pittayapat P., Bornstein M.M., Imada T.S., Coucke W., Lambrichts I., Jacobs R. (2015). Accuracy of linear measurements using three imaging modalities: Two lateral cephalograms and one 3D model from CBCT data. Eur. J. Orthod..

[23] Quintero J.C., Trosien A., Hatcher D., Kapila S. (1999). Craniofacial imaging in orthodontics: Historical perspective, current status, and future developments. Angle Orthod..

[24] Baumrind S., Frantz R.C. (1971). The reliability of head film measurements. 2. Conventional angular and linear measures. Am. J. Orthod..

[25] Baumrind S., Frantz R.C. (1971). The reliability of head film measurements. 1. Landmark identification. Am. J. Orthod..

[26] Arai Y., Tammisalo E., Iwai K., Hashimoto K., Shinoda K. (1999). Development of a compact computed tomographic apparatus for dental use. Dentomaxillofac. Radiol..

[27] Mozzo P., Procacci C., Tacconi A., Martini P.T., Andreis I.A. (1998). A new volumetric CT machine for dental imaging based on the cone-beam technique: Preliminary results. Eur. Radiol..

[28] Cattaneo P.M., Melsen B. (2008). The use of cone-beam computed tomography in an orthodontic department in between research and daily clinic. World J. Orthod..

[29] de Oliveira A.E., Cevidanes L.H., Phillips C., Motta A., Burke B., Tyndall D. (2009). Observer reliability of three-dimensional cephalometric landmark identification on cone-beam computerized tomography. Oral Surg. Oral Med. Oral Pathol. Oral Radiol. Endod..

[30] Cattaneo P.M., Yung A.K.C., Holm A., Mashaly O.M., Cornelis M.A. (2019). 3D landmarks of Craniofacial Imaging and subsequent considerations on superimpositions in orthodontics-The Aarhus perspective. Orthod. Craniofac Res..

[31] Yildirim E., Karacay S., Erkan M. (2014). Condylar response to functional therapy with Twin-Block as shown by cone-beam computed tomography. Angle Orthod..

[32] Elfeky H.Y., Fayed M.S., Alhammadi M.S., Soliman S.A.Z., El Boghdadi D.M. (2018). Three-dimensional skeletal, dentoalveolar and temporomandibular joint changes produced by Twin Block functional appliance. J. Orofac. Orthop..

[33] Jiang Y.Y., Sun L., Wang H., Zhao C.Y., Zhang W.B. (2020). Three-dimensional cone beam computed tomography analysis of temporomandibular joint response to the Twin-block functional appliance. Korean J. Orthod..

[34] Björk A. (1947). The face in profile: An anthropological X-ray investigation on Swedish children and conscripts. Svensk Tandläkare-Tidskrift.

[35] Ludlow J.B., Timothy R., Walker C., Hunter R., Benavides E., Samuelson D.B., Scheske M.J. (2015). Effective dose of dental CBCT-a meta analysis of published data and additional data for nine CBCT units. Dentomaxillofac. Radiol..

[36] Kapila S.D., Nervina J.M. (2015). CBCT in orthodontics: Assessment of treatment outcomes and indications for its use. Dentomaxillofac. Radiol..

[37] LeCornu M., Cevidanes L.H., Zhu H., Wu C.D., Larson B., Nguyen T. (2013). Three-dimensional treatment outcomes in Class II patients treated with the Herbst appliance: A pilot study. Am. J. Orthod. Dentofac. Orthop..

[38] Nelson B., Hansen K., Hagg U. (2000). Class II correction in patients treated with class II elastics and with fixed functional appliances: A comparative study. Am. J. Orthod. Dentofac. Orthop..

[39] Wei R.Y., Atresh A., Ruellas A., Cevidanes L.H.S., Nguyen T., Larson B.E., Mangum J.E., Manton D.J., Schneider P.M. (2020). Three-dimensional condylar changes from Herbst appliance and multibracket treatment: A comparison with matched Class II elastics. Am. J. Orthod. Dentofac. Orthop..

[40] Antonarakis G.S., Kiliaridis S. (2007). Short-term anteroposterior treatment effects of functional appliances and extraoral traction on class II malocclusion. A meta-analysis. Angle Orthod..

[41] Tulloch J.F., Phillips C., Koch G., Proffit W.R. (1997). The effect of early intervention on skeletal pattern in Class II malocclusion: A randomized clinical trial. Am. J. Orthod. Dentofac. Orthop..

[42] Baysal A., Uysal T. (2014). Dentoskeletal effects of Twin Block and Herbst appliances in patients with Class II division 1 mandibular retrognathy. Eur. J. Orthod..

[43] Siara-Olds N.J., Pangrazio-Kulbersh V., Berger J., Bayirli B. (2010). Long-term dentoskeletal changes with the Bionator, Herbst, Twin Block, and MARA functional appliances. Angle Orthod..

[44] Mills C.M., McCulloch K.J. (2000). Posttreatment changes after successful correction of Class II malocclusions with the twin block appliance. Am. J. Orthod. Dentofac. Orthop..

[45] Faltin K.J., Faltin R.M., Baccetti T., Franchi L., Ghiozzi B., McNamara J.A. (2003). Long-term effectiveness and treatment timing for Bionator therapy. Angle Orthod..

[46] O’Brien K., Wright J., Conboy F., Sanjie Y., Mandall N., Chadwick S., Connolly I., Cook P., Birnie D., Hammond M. (2003). Effectiveness of early orthodontic treatment with the Twin-block appliance: A multicenter, randomized, controlled trial. Part 1: Dental and skeletal effects. Am. J. Orthod. Dentofac. Orthop..

[47] Perillo L., Femiano A., Palumbo S., Contardo L., Perinetti G. (2013). Skeletal and dental effects produced by functional regulator-2 in pre-pubertal class II patients: A controlled study. Progress. Orthod..

[48] Ehsani S., Nebbe B., Normando D., Lagravere M.O., Flores-Mir C. (2015). Short-term treatment effects produced by the Twin-block appliance: A systematic review and meta-analysis. Eur. J. Orthod..

